# Geographic and topographic determinants of local FMD transmission applied to the 2001 UK FMD epidemic

**DOI:** 10.1186/1746-6148-4-40

**Published:** 2008-10-03

**Authors:** Paul R Bessell, Darren J Shaw, Nicholas J Savill, Mark EJ Woolhouse

**Affiliations:** 1 Centre for Infectious Diseases, School of Biological Sciences, University of Edinburgh, Kings Buildings, West Mains Road, Edinburgh, EH9 3JT, UK; 2 Veterinary Clinical Sciences, R(D)SVS, University of Edinburgh, Easter Bush Veterinary Centre, Roslin, Midlothian, EH25 9RG, UK

## Abstract

**Background:**

Models of Foot and Mouth Disease (FMD) transmission have assumed a homogeneous landscape across which Euclidean distance is a suitable measure of the spatial dependency of transmission. This paper investigated features of the landscape and their impact on transmission during the period of predominantly local spread which followed the implementation of the national movement ban during the 2001 UK FMD epidemic. In this study 113 farms diagnosed with FMD which had a known source of infection within 3 km (*cases*) were matched to 188 *control *farms which were either uninfected or infected at a later timepoint. *Cases *were matched to *controls *by Euclidean distance to the source of infection and farm size. Intervening geographical features and connectivity between the source of infection and *case *and *controls *were compared.

**Results:**

Road distance between holdings, access to holdings, presence of forest, elevation change between holdings and the presence of intervening roads had no impact on the risk of local FMD transmission (p > 0.2). However the presence of linear features in the form of rivers and railways acted as barriers to FMD transmission (odds ratio = 0.507, 95% CIs = 0.297,0.887, p = 0.018).

**Conclusion:**

This paper demonstrated that although FMD spread can generally be modelled using Euclidean distance and numbers of animals on susceptible holdings, the presence of rivers and railways has an additional protective effect reducing the probability of transmission between holdings.

## Background

Foot and Mouth Disease (FMD) is a highly infectious viral disease of cloven hoofed animals. The outbreak that occurred in the UK between February and September 2001 resulted in 2026 Infected Premises (IPs) on the British mainland. An estimated 4.2 million animals were slaughtered for disease control purposes and another 2.5 million for welfare purposes [1]. The estimated direct and indirect costs of the epidemic were £6 billion [2].

The FMD virus can be transmitted through a variety of routes including aerosol transmission, direct contact between animals and on fomites, furthermore, the virus has been shown to spread over distances greater than 100 km by viral plumes [3]. During the early stages of the 2001 UK epidemic long range transmission events were facilitated by the movements of infected animals; this was largely brought to an end by the imposition of the national movement ban (NMB) on susceptible animals introduced on the 23rd February. From this date, disease transmission was more localised with over 50% of transmission events occurring across distances of less than 3 km and more than 80% over distances of less than 10 km [4-6]. Some mathematical models of FMD transmission [6-8] incorporate this distance decay effect in the form of a kernel which describes the declining probability of infection with distance from an infectious source. This kernel assumes that the landscape is homogeneous and that elements of the landscape such as roads, rivers and topography have no influence on the likelihood of virus transmission. Furthermore, the kernel assumes that the distance between two holdings can be adequately modelled as straight line Euclidean distance rather than road distance or topographically adjusted Euclidean distance.

Savill et al [9] tested the utility of Euclidean distance compared to road distance between holding pairs at a coarse scale. The authors calculated Euclidean distances and road distances between an IP and a potential 'daughter IP' and between IPs and susceptible non-IPs over distances of up to 10 km. They found no statistically significant difference in the correlations for road to Euclidean distance between the two groups of holdings where they were not separated by river estuaries. However, where pairs of holdings were separated by a river estuary (in this case the Severn estuary and Solway Firth) there was a significant difference. This is because road travel across estuaries is limited by the availability of crossing points, so road distance is generally greater when farms were separated by an estuary compared to instances in which they are not. In these instances road distance becomes a more appropriate measure of transmission risk. However, the analysis of Savill et al [9] considered all possible infection events within 10 km, as opposed to having used transmission tracing data gathered through epidemiological investigations during the epidemic. Therefore the effects of road distance on transmission may have been masked by the nature of the IP pairings being used.

The current paper will extend the work of Savill et al [9] by investigating the potential for geographical features to act as conduits or barriers to infection during the period of local spread following the NMB. The analysis will be conducted at a more localised scale and at a far greater precision by analysing only known infection events and by looking at a greater range of geographical features. This will be accomplished by considering distance matched *source-case-control *groups where the *source *was believed to have infected the *case *but did not infect the *control*. The geographical features separating *source-case *and *source-control *pairings will then be compared.

## Results and discussion

In the univariate analysis most predictor variables were not associated with differences between *cases *and *controls *at the p < 0.2 level, the exceptions were rivers and railways and road distance for which p = 0.088, 0.166 and 0.098 respectively (Table 1). Numbers in the present groups for both rivers and railways were relatively small (61 and 27 respectively, Table 2). These features prevent transmission by forming linear barriers which inhibit movements of humans and animals across them, features described by Smith et al [10] as *semipermeable barriers*. Therefore, the rivers and railways categories were merged to form a single barriers variable and this was statistically significant at the p < 0.05 level (p = 0.018), with the number of *cases *separated by a barrier significantly lower than the number of *controls *(Table 3). The odds ratio of there being a barrier between *source *and *case/control *holding were 0.5 as high for *cases *than for *controls *(odds ratio = 0.507, 95% CI = 0.297, 0.887).

**Table 1:** Univariate generalised linear mixed model (binomial errors) analysis of predictor variables.

Variable	Unit	odds ratio (95% CIs)	t-value	p
Road distance	km	1.507 (0.93,2.44)	1.66	0.098
Rivers	Absent	1	-	-
	Present	0.594 (0.33, 1.08)	-1.72	0.087
Rail	Absent	1	-	-
	Present	0.550 (0.24, 1.28)	-1.389	0.166
Forest	Absent	1	-	-
	Present	1.286 (0.69, 2.40)	0.790	0.431
Road	0	1	-	-
	1	0.959 (0.46, 2.02)	-1.109	0.913
	2 & 3	0.737 (0.33, 1.67)	-0.732	0.465
Access	1	1	-	-
	2	1.17 (0.72,1.90)	0.614	0.540
	3	0.951 (0.41,2.19)	-0.118	0.906
Elevation change	$\sqrt{m}$	0.965 (0.92, 1.01)	-1.448	0.149

**Table 2:** Number of instances of holdings being separated by rivers and railways.

		Absent	Present
River	Case	94 (83.2)	16 (14.2)
	Control	141 (74.6)	45 (23.8)

Rail	Case	105 (92.9)	7 (6.2)
	Control	166 (87.8)	20 (10.6)

River & Rail	Case	91 (80.5)	22 (19.5)
	Control	128 (67.7)	61 (32.3)

Percentages based upon the row totals are in brackets.

**Table 3:** Multivariate generalised linear mixed model (binomial error term) of barriers as a risk factor for risk of FMD transmission.

Predictor	Unit	odds ratio (95% CIs)	t-value	p-value
Intercept	-		-2.480	0.014
Barriers	No	1	-	-
	Yes	0.507 (0.297,0.887)	-2.382	0.018

In addition to controlling for Euclidean distance, animal numbers and indirectly (by capping the distance at 3 km) for Disease Control Centre (DCC) in the *group *selection process these risk factors were further checked by inserting each of these terms into the model as a covariate. The insertion of these terms did not affect the significance of the barriers term, however the insertion of the Euclidean distance term did alter the p-value of the road distance term such that with the Euclidean distance term the p-value of the road distance term is greater than 0.5.

The presence of linear features in the form of rivers or railways is the only geographical influence on FMD transmission which could be detected using this methodology. The principal mechanisms by which linear features reduce transmission would be by the prevention of the movements of animals, people and vehicles. Two farms separated by a small river may be less likely to have contacts, exchange personnel and have animals coming into contact compared to two farms separated by a fence or open farmland. A similar effect has been observed in rabies transmission, in which the presence of rivers were found to reduce the rate of progression of waves of rabies to one seventh of its speed before the river [10].

Although rivers and railways together were a statistically significant barrier to FMD transmission, roads were not a statistically significant barrier to transmission (Table 1). This supports previous studies [9] which demonstrated that even a major road such at the M6 motorway did not act as a barrier to FMD transmission. This may be a result of roads forming a more permeable barrier than rivers and railways. A set of 325 *cases *and 766 *controls *which had not been controlled for animal numbers was analysed using a generalised linear model with binomial error term to evaluate any differences in terms of numbers of animals on *cases *and *controls *(Table 4). Numbers of both cattle and sheep were statistically significantly lower on *controls *compared to *cases *irrespective of data source used (see methods). This supports previous findings that showed that IPs were larger farms [6] and also justified the criteria of selecting *controls *on animal numbers.

**Table 4:** Univariate generalised linear model (binomial error term) analysis of animal numbers by species on preliminary *cases *compared to *controls*.

Species	Case data (n)	Control data (n)	Estimate	SE	χ^2^	p
Cattle (100s)	DCS (325)	Census (766)	-0.561	0.061	98.59	<0.001
Cattle (100s)	Census (218)	Census (766)	-0.626	0.080	63.34	<0.001

Sheep (1000s)	DCS (325)	Census (766)	-0.998	0.153	46.7	<0.001
Sheep (1000s)	Census (218)	Census (766)	-0.762	0.161	21.24	<0.001

These detailed analyses of geographical influences on virus transmission were made possible by the highly detailed FMD datasets. The identification of *source-case *pairings was based upon the identification of putative sources of infection for IPs during the epidemic. These sources were identified through epidemiological investigations; data which have been used in previous studies [6,11]. However for many IPs the source could not be identified with certainty. Analysis of these data identified 386 inconsistencies in source identification based upon dates of infection and slaughter. Although these IPs were excluded, there is no simple way to detect any remaining errors. Since the epidemic it has been demonstrated that sources of infection can be established with a high degree of certainty using FMDV sequence data [12], however, these techniques have only been applied to 22 IPs from 2001 and therefore are of little value for this study.

## Conclusion

Amongst the mainly geographical risk factors studied here, Euclidean distance and the number of animals on the susceptible holding appeared to be the most important determinant of risk of FMD transmission, a conclusion supported by previous analysis [6,9]. Beyond this it is difficult to draw further distinctions between holdings in terms of their relative susceptibilities based on these analyses. Despite the fact that some infectious contacts will be facilitated by the road network, road distance has again been shown not to be a more accurate predictor of transmission risk than simple Euclidean distance. However, this paper has demonstrated that these infectious contacts can be reduced by the presence of linear barriers in the form of rivers and railways but not roads, possibly because roads are too permeable to contacts across them. While the analysis was conducted on a small subset of 113 IPs (5.8% of the IPs infected after the NMB), the size of the subset was the result of the rigorous *group *selection procedure. These IPs were compared to all IPs infected after the NMB to ensure that they were a representative sample and although almost significantly different in terms of species composition and spatial distribution the *cases *were a representative subset of all other IPs. In conclusion, the presence of rivers and railways should be incorporated into spatial models of FMD transmission to give more accurate estimates of transmission risk.

## Methods

The study used a case-control methodology in which *case *and *control *selection was based upon their geographical relationship to a *source *IP. The process of matching *controls *to *cases *and *sources *is described below.

### Premises data

*Sources *and *cases *were selected from the 2026 Infected Premises (IPs). Data on IPs are stored in the DEFRA Disease Control System (DCS) database [13]. This dataset included a coordinate for the main farm building, numbers of animals culled on the holding, estimated date of infection, dates on which the IP was reported and slaughtered and the most likely source of infection for many IPs. The coordinates recorded in the DCS were the coordinates of the main farm building as this has been identified as the best location from which to georeference farm holdings [14]. However, in the DCS, instances in which the infected animals are more than 1 km from the main holding were georeferenced to the location of the affected stock [13].

IPs were considered as potential *cases *if they were:

1. Estimated to have been infected after the NMB. This is because before the NMB was imposed there were a greater range of mechanisms of spread of FMD operating.

2. The IP had a reliable source of infection identified. A source of infection was considered reliable if the daughter IPs' livestock was infected after the source IP was infected but before the source IP was slaughtered.

3. The IP had a source of infection within 3 km.

*Controls *were selected from the 139,195 holdings registered on the June 2000 agricultural censuses of England, Scotland and Wales. There was no census conducted in 2001, so the June 2000 census was the most appropriate dataset for these analyses and has been used extensively in analysis of the 2001 FMD epidemic [4,7,15]. Data in the agricultural census includes a coordinate for the main farm building and animal numbers by species.

A holding was included as a potential *control *if it:

1. Had no off-fields. Holdings with off-fields were identified using the holding number of the parcel of land and the name of the owner (data not shown). The number of animals on the off-fields can not be known with certainty and therefore only holdings without off-fields were included in these analyses.

2. Was within 4 km of an IP. A distance of 4 km was used as this allowed a window either side of the 3 km definition of local spread.

### Identifying groups

Potential *controls *were matched to a *case-source *pair based on the Euclidean distance from *source *to potential *control *(*d*_*n*_) and the distance from *source *to *case *(*d*_*c*_). Groups were matched where:

(1) -250*m *<*d*_*n *_- *d*_*c *_< 199.5*m*

Asymmetric bounds were used to equalise the area either side of *d*_*c*_, thus ensuring that similar numbers of *controls *were selected on either side of *d*_*c*_.

*Controls *which were culled at a later point in the epidemic were included if the animals on the premises were slaughtered more than 14 days after the *source *was slaughtered. This allows for the maximum incubation time [16,17] removing the possibility that the *control *had been infected by the *source*. Furthermore, if the potential *control *was an IP it must have been infected at least 14 days after the *source *was slaughtered to allow for error in the estimated infection date of the potential *control*.

**Figure 1:**
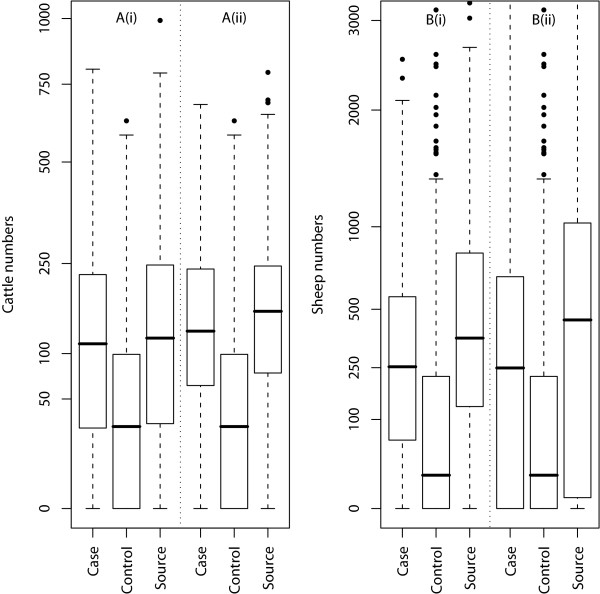
**Comparisons of numbers of cattle (A) and sheep (B) on *cases*, *sources *and *controls *broken down by census animal numbers (ii) and DCS animal numbers (i).** Whiskers represent 1.5 times the standard deviation and the y-axes are square-root transformed.

It has previously been shown [6] that animal numbers on the premises are a risk factor for susceptibility. Therefore groups were evaluated to look for differences between *case *and *control*(*s*) in terms of animal numbers (Figure 1). As a consequence of differences in the definition of a farm premises described above and the time of year at which the data were gathered, animal numbers on the agricultural census and DCS may have differed for an individual holding. Therefore, animal numbers on *case *and *control*(*s*) were compared using two methods depending upon data availability:

1. For the *cases *for which there is census data, the census numbers on the *case *were compared to the census numbers on the *control*.

2. Animal numbers from the DCS were compared to animal numbers on the census for all *case-control *pairs.

To ensure that the number of animals was controlled for, the numbers of animals on the *control *had to be similar to those on the *case *as defined by the following criteria:

1. The *control *had at least 70% of the numbers of cattle, sheep and pigs on the *case*.

2. The *control *had no more than 50 fewer cattle than the *case *and no more than 100 fewer sheep and pigs than the *case*.

3. The *control *must have held cattle if the *case *held cattle.

The latter criterion made allowances for relatively small holdings, for instance it allowed a *case *with 50 head of cattle to be paired with a *control *with 30 head.

There were instances of a *control *being a *control *for 2 *cases*. In these instances the *controls *was assigned to just one *case*; if one of the *cases *had more than one *control *the *control *was removed from that group, otherwise one of the groups was selected at random. The resulting dataset comprised 113 groups which were composed of 113 *cases *and 188 *controls*. The selection criteria which generated these data are summarised as:

1. The *control *if culled was culled more than 14 days after the *case*.

2. The *control *if an IP was infected more than 14 days after the *case*.

3. Groups which meet the distance matching criteria described above.

4. Matching for animal numbers as described above.

5. Ensuring that a *control *was matched to only one *case*.

The 113 *cases *were checked for representativeness by comparing the *cases *to all other IPs infected after the NMB. The *cases *were not statistically significantly different from the IPs in terms of species composition described by whether the holding was cattle only, sheep only or mixed ($\chi_{2}^{2}$ = 5.59 p = 0.061), temporal distribution (Kolmogorov-Smirnov test based upon estimated dates of infection p = 0.126) and spatial distribution based upon Disease Control Centre (Fishers Exact Test p = 0.057). Therefore the identified *cases *appeared to be a valid representation of the IP set.

### Predictors

Road distance was calculated between two points by overlaying the data onto scanned Ordnance Survey 1:50,000 scale topographic maps supplied by the EDiNA Digimap service [18] and calculating the shortest distance along roads using the measure tool in ESRI ArcGIS 9. Intervening rivers and railways and forest were derived from the same maps in turn by taking a straight line between two points and evaluating intervening features by presence or absence of the features. Intervening roads were measured on the following scale:

0 = no intervening road.

1 = Minor road only.

2 = B, A road or motorway.

Accessibility was derived from the locations of *cases *and *controls *on the maps on the following scale:

1 = Track or dead end road.

2 = Beside a minor road

3 = Beside a main road.

Cumulative elevation change between two holdings was derived from an OS 1:50,000 Digital Elevation Model supplied by EDiNA [18] after tiles for the necessary regions were mosaiced in ArcGIS 9.0. Straight lines between the points were generated in ESRI ArcView 3.2 and cumulative elevation change along the lines calculated using the surface tools extension in ArcView 3.2 [19].

### Statistical analysis

The outcome for each holding (whether it was a *case *or *control*) formed a binary outcome variable with the *control *category the reference level. The effect of having multiple distance matched *controls *for each *case *was handled by classifying each *case *and all its *controls *as a group. The group was added to the logistic regression model as a random effect to form a mixed effects logistic regression model in which variables were considered statistically significant when p < 0.05. This was implemented in the MASS Package [20] for the R statistical environment [21]. Interrelationships between univariate predictors which were significant at p < 0.2 were analysed in a multivariate model. Significance of the predictors was evaluated using the Wald statistic and the logits of continuous variables were inspected for linearity. The effectiveness of the selection process was checked by inserting Euclidean distance (*source *to *case*), animal numbers by species on the holding and DCC into the model. Any major change to the significance of predictor would suggest that a factor had not been adequately controlled for and could bias the results.

## Authors' contributions

PRB drafted the manuscript. PRB, DJS, NJS and MEJW conceived the study. DJS helped with statistical analysis and with cleaning the data. All authors critically assessed the study and read and approved the final manuscript.
